# Effect of family medicine groups on visits to the emergency department among diabetic patients in Quebec between 2000 and 2011: a population-based segmented regression analysis

**DOI:** 10.1186/s12875-016-0422-2

**Published:** 2016-02-29

**Authors:** Renee Carter, Amélie Quesnel-Vallée, Céline Plante, Philippe Gamache, Jean-Frédéric Lévesque

**Affiliations:** Department of Epidemiology, Biostatistics and Occupational Health, Faculty of Medicine, McGill University, 1020 Pine Avenue West, Montreal, Quebec Canada; Department of Sociology, Faculty of Arts, McGill University, 855 Sherbrooke Street West, Montreal, Quebec Canada; Institut National de Santé Publique du Québec, 945 Avenue Wolfe, Ste-Foy, Québec, Canada; Bureau of Health Information, Level 11 Sage Building, 67 Albert Avenue, Chastwood, NSW Australia; Centre for Primary Health Care and Equity, University of New South Wales, Level 3, AGSM Building, Botany Street Gate 11, Sydney, NSW 2052 Australia

**Keywords:** Family medicine groups, Quebec, Diabetes, Primary care, Emergency department

## Abstract

**Background:**

Family Medicine Groups (FMG) were introduced in Quebec in 2002 to re-organize primary care practices and encourage inter-professional service delivery. We measured visits to the emergency department (ED) for acute complications related to diabetes as a proxy for access to and quality of primary care, before and after the reform using an open cohort of individuals diagnosed with type 1 and type 2 diabetes.

**Methods:**

The weekly rate of ED visits between April 1, 2000 and March 31, 2012 were derived from administrative databases. We performed an interrupted segmented regression analysis to obtain the estimated and predicted rates of visits in the years following the introduction of the reform. An outcome control series of diabetic patients visiting the ED to treat appendicitis was incorporated to strengthen the study’s internal validity.

**Results:**

After 9 years of reform implementation, we observed a statistically significant absolute decrease of 2.12 and 2.25 ED visits per 10,000 diabetic patients per week to treat acute diabetes-related complications in urban and rural areas, respectively. However, the magnitude of the changes between the estimated and predicted rates did not differ significantly over time. No statistically significant change in the rate of ED visits for appendicitis was observed.

**Conclusion:**

Our findings suggest that the introduction of the FMG model produced reductions in the weekly rate of avoidable visits to the ED. Our results also imply that despite a greater proportion of the diabetes population being enrolled with FMG physicians across the province over time, the added benefit may be minimal. More studies examining this issue are needed to inform future policy.

## Background

In 2000, Quebec’s Clair Commission called for the re-organization of primary care to place an emphasis on population health, in addition to continuity of and accessibility to care [1]. Since 2002, Family Medicine Groups (FMG) have been implemented across the province by physicians. The FMG model for primary care was introduced with the aim to promote patient enrolment with a primary care physician and establish inter-professional team-based service delivery. The FMG model was taken up to varying degrees in all regions across the province with greater implementation occurring in rural areas during the early years of the reform [2]. The practices are intended to include six to ten physicians and two nurses to serve a population of 10,000 to 20,000 patients. Ideally, nurses alleviate physician caseloads by seeing patients whose concerns do not require physician expertise. The presence of allied health professionals such as pharmacists, physiotherapists or social workers, is not a program requirement and accordingly varies by practice [3]. Government financial incentives are offered to support administrative staff and nurse salaries, and modernize practices through information technology improvements [4]. In return, practices must guarantee greater access to care through longer practice hours on weekdays, weekends, and holidays, in addition to on-call services for vulnerable patients [4, 5]. Increasing accessibility to and continuity of primary care have been identified by the Commissioner of Health and Welfare as health system priorities and FMG practices are considered the vehicle for achieving these goals [6].

The rise in the prevalence of diabetes over the last two decades has made it a priority for chronic disease surveillance in Quebec [7, 8]. As it is estimated that nearly 80 % of diabetes care takes place in primary care settings, access to and quality of these services is paramount in optimal disease management [9, 10]. Indeed, glycemic control among type 1 and type 2 diabetes patients is associated with a lowered risk of acute and long term adverse outcomes [11, 12]. Thus, although patient self-care remains key to reducing the risk of complications, recent evidence suggests that integrated models of primary care service delivery, such as FMGs, are better tailored to supporting patients and providers in chronic disease management [10].

In the context of an aging population, it is of relevance to policy makers to determine whether reforms to primary care have changed access to and quality of care among those with chronic ambulatory care sensitive conditions (ACSC) that are largely managed by family physicians. Our objective was to examine the change in the rate of visits to the ED produced by the FMG model within the province’s diabetic patient population.

## Methods

### Study design

We conducted a population-based retrospective study using a segmented regression analysis of an interrupted time series. The segmented regression analysis is useful for determining whether the reform produced any changes in the outcome, and if so, whether these changes were abrupt or gradual [13]. The implementation of the FMG model began in November 2002 [14] and was treated as an ‘interruption’ in the time series that distinguished between the pre and post reform periods. To strengthen our design, we incorporated a control series for an outcome that we anticipated would not be correlated with the FMG reform but that would respond in similar ways as our main outcome series to contextually relevant threats to the study’s validity. Accordingly, we selected visits to the ED among diabetic patients for appendicitis since this is a non-ACSC that should not be associated with the delivery of primary care [15] yet is still sensitive to changes in the health system that are also related to our main outcome series.

### Data source

Data from the Quebec Integrated Chronic Disease Surveillance System (QICDSS) were used for the development of healthcare utilization indicators [16]. The analysis covered 12 fiscal years from April 1, 2000 to March 31, 2012. The datasets were linked using scrambled identification numbers and cases of diabetes (excluding gestational diabetes) were identified from the medical claims and hospital admissions data using an algorithm previously validated in a Canadian study [17, 18]. We based our regional analyses on the health and social services territorial classification system which groups 18 regions into four categories (university, peripheral, intermediary and remote) according to their proximity to urban centers [19]. Observations from four health and social services regions were excluded because service provision in these areas is either structurally different from those of the rest of province or due to the tendency for patients seek care across the border in Ontario thereby producing unreliable annual estimates of health service utilization. Accounting for these exclusions, the number of individuals diagnosed with diabetes ranged from 275,728 in 2000 to 533,438 in 2011.

### Outcome measurement

We defined the rate of ED visits for acute diabetes-related complications and appendicitis among patients aged 20 and over as our outcomes of interest. We selected ICD-9 codes based on the Canadian Institute of Health Information’s list of ambulatory care sensitive codes specific to diabetes (eg.: hyperglycemic and hypoglycemic emergencies) [20]. ED visits occurring between April 1, 2000 and March 31, 2012 were used in the analysis. A previously validated algorithm to identify distinct visits to the ED in Quebec was applied to the medical claims data [21]. Using the QICDSS, we calculated mid-year population estimates of the diabetes population in Quebec to derive the denominator for the rates.

### Statistical analysis

The time series was produced from daily ED records aggregated to the number of visits per week for each outcome. This produced 626 weeks (observations) in our time series, which is consistent with the number of weeks between April 1, 2000 and March 31, 2012. We fit a regression line to each segment of the series (before and after November 2002) using a negative-binomial distribution and the log of the mid-year diabetic patient population as the offset term. Our model included an intervention variable and a variable denoting time since the intervention (number of weeks). We included demographic covariates measured at the province-year level: average age and sex. We included fixed effect terms for year and season to control for secular trends in the rate of avoidable visits to the ED since long-term temporal changes may be correlated with the FMG practice model. For instance, fixed effects for year would control for secular trends in the family physician labor force if there were a growing preference for practicing part-time. We also included a linear time variable to address residual confounding due to unmeasured regional characteristics that could produce gradual trends in the rate of avoidable visits to the ED. The Durbin-Watson test revealed no significant autocorrelation in the data and robust standard errors were specified for all models.

The literature recommends at least 12 data points before and after the intervention, and at least 100 observations making up each data point, in order to have sufficient statistical power to detect intervention effects [13]. Accordingly, we grouped the four regional classifications into two categories that we refer to as urban and rural for our main outcome series. Separate regression analyses were conducted for each region. We performed a single regression analysis for our control series since the rate of visits to the ED to treat appendicitis is not expected to systematically differ by region. We contrasted the estimated rate of ED visits from the model with the extrapolated rate of ED visits, had the reform not occurred, according to the amount of time elapsed since the reform was introduced. The effect of the reform was computed 3, 6, and 9 years after implementation, indicated by *T*_*3*_, *T*_*6*_ and *T*_*9*_, respectively, in Figs. 2, 3 and 4. For each time point, the extrapolated rate was subtracted from the model estimate of the rate of ED visits: *Ŷ*_*t* (*with reform*)_ − *Ŷ*_*t* (*without reform*)_ [13]. We reported results on the additive scale and, therefore, estimates with negative values indicate a reduction in the rate of avoidable ED visits. We expressed the magnitude of the effect as risk differences with 95 % confidence intervals using the Satterthwaite approximation to calculate the standard errors. A confidence interval that does not include zero indicates a statistically significant effect at the *p* = 0.05 level. Analyses were performed using SAS version 9.3.

### Ethics review

Government bodies in legal possession of the databases, in addition to Quebec’s Comité d’éthique de santé publique and the Commission d’accès à l’information du Québec, have approved the creation of the QICDSS and its use for chronic disease surveillance. The creation of the QICDSS and access to its data meet stringent standards of security and privacy. This study is part of a doctoral project that was approved by the Faculty of Medicine’s Institutional Review Board at McGill University. Individual participant consent was not required in accordance with the Canadian Tri-Council Policy Statement: Ethical Conduct for Research Involving Humans.

## Results

**Fig. 1:**
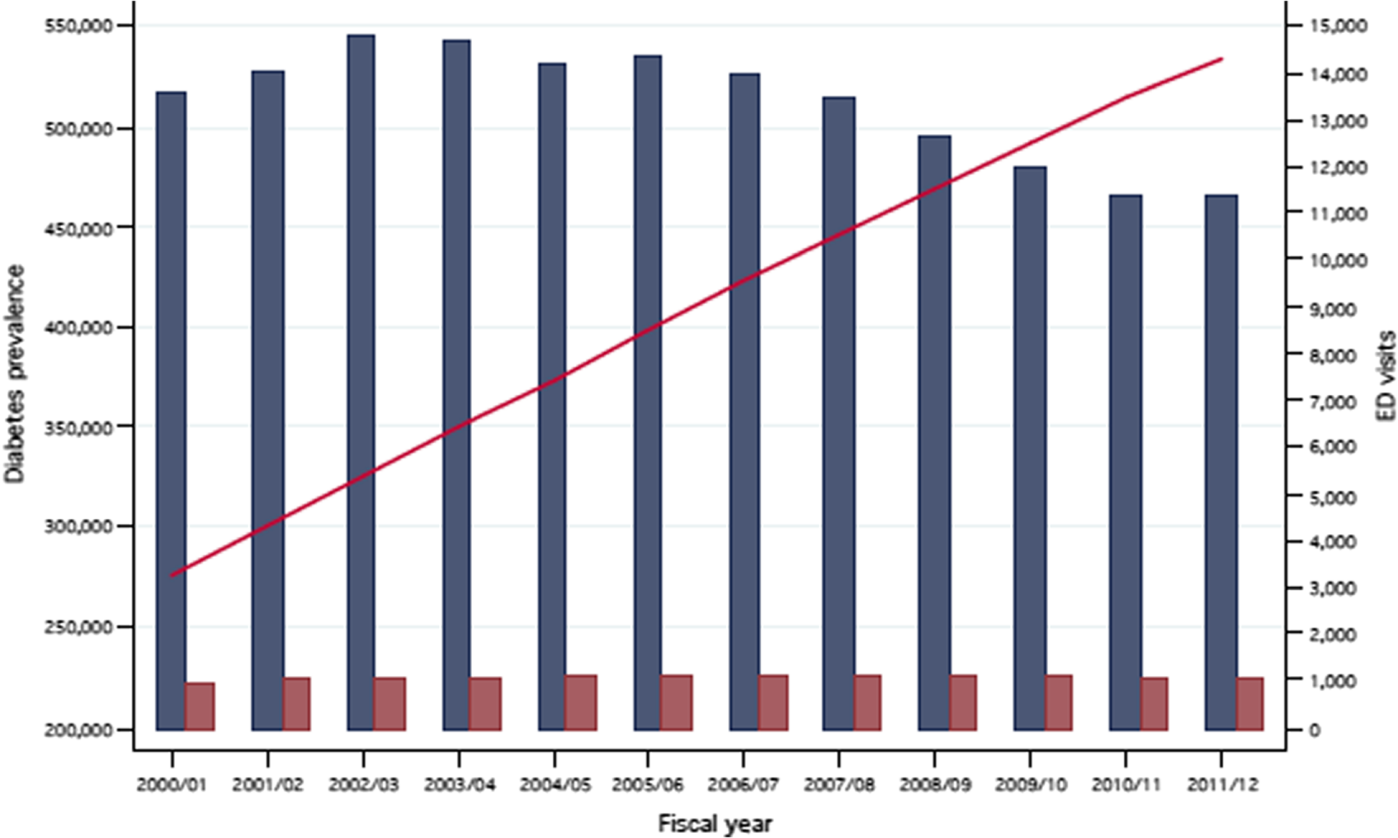
Diabetes prevalence and number of emergency department visits (avoidable and appendicitis) in Quebec, 2000/01 to 2011/12. Legend: *Blue bar* = ED visits (avoidable). *Red bar* = ED visits (appendicitis). *Line* = Diabetes prevalence

Figure 1 illustrates the relationship between the prevalence of diabetes in Quebec and the number of ED visits by fiscal year. The prevalence of diabetes was steadily increasing over the study period while the number of avoidable visits to the ED was on the decline. The number of visits to the ED for appendicitis remained stable over the follow-up period.

Table 1 presents results from the regression models. In urban regions, the model estimates a non-significant change in the level immediately following the reform (RR = 1.04; 95 % CI = 0.98, 1.10). The change in trend in the weeks following the reform was also non-significant (RR = 0.99; 95 % CI = 0.99, 1.00). Results from the model for rural regions indicated a statistically significant increase in the rate of avoidable visits immediately following the introduction of the reform (RR = 1.12; 95 % CI = 1.04, 1.20) and a non-significant change in the trend (RR = 0.99; 95 % CI = 0.99, 1.00). The final column contains the estimates for the effect of the reform, calculated from the linear combination of coefficients and expressed as the post-intervention slope (β_1_ + β_3_). The results indicate that the FMG model produced a significant 1 % decrease in the post-reform rate of avoidable visits to the ED per 10,000 diabetic patients per week (RR = 0.99; 95 % CI = 0.98, 0.99). Similarly to the urban regions, a significant reduction of 1 % in the rate of avoidable visits to the ED per 10,000 diabetic patients per week was found in the post-reform period. Per annual quarter, this represents a 3 % reduction in the number of avoidable visits to the ED per 10,000 diabetic patients. The appendicitis control series indicated no statistically significant effect of the reform (RR = 1.00; 95 % CI = 0.99, 1.01). Model estimates were robust to intervention lag terms and control for potential wild data points.

**Table 1:** Model results for the effect of the FMG reform on visits to the ED among diabetic patients (avoidable and appendicitis)

Outcome	Intercept (β_0_)	Baseline trend (β_1_)	Level change (β_2_)	Change in trend (β_3_)	Post-reform trend (β_1_ + β_3_)
	Number of visits per 10,000 diabetic patients at baseline	Trend in the number of visits per 10,000 diabetic patients per week before the reform	Level change in the number of visits per 10,000 diabetic patients immediately following the reform	Trend change in the number of visits per 10,000 diabetic patients per week following the reform	Trend in the number of visits per 10,000 diabetic patients per week after the reform
		(95 % CI)	(95 % CI)	(95 % CI)	(95 % CI)
Avoidable visits (urban areas)	9.49	0.99 (0.99, 1.00)	1.04 (0.98, 1.10)	0.99 (0.99, 1.00)	0.99 (0.98, 0.99)
		*p* = 0.07	*p* = 0.11	*p* = 0.10	*p* < 0.05
Avoidable visits (rural areas)	10.31	0.99 (0.99, 1.00)	1.12 (1.04, 1.20)	0.99 (0.99, 1.00)	0.99 (0.98, 0.99)
		*p* = 0.78	*p* < 0.05	*p* = 0.21	*p* < 0.05
Appendicitis	0.66	1.00 (0.99, 1.01)	0.97 (−0.84, 1.12)	1.00 (0.99, 1.00)	1.00 (0.99, 1.01)
		*p* = 0.29	*p* = 0.72	*p* = 0.81	*p* = 0.28

Estimates are conveyed as rate ratios

Final models included: time, FMG reform, post-FMG variable, dummy variables for seasonal quarter, and dummy variables for fiscal year

**Fig. 2:**
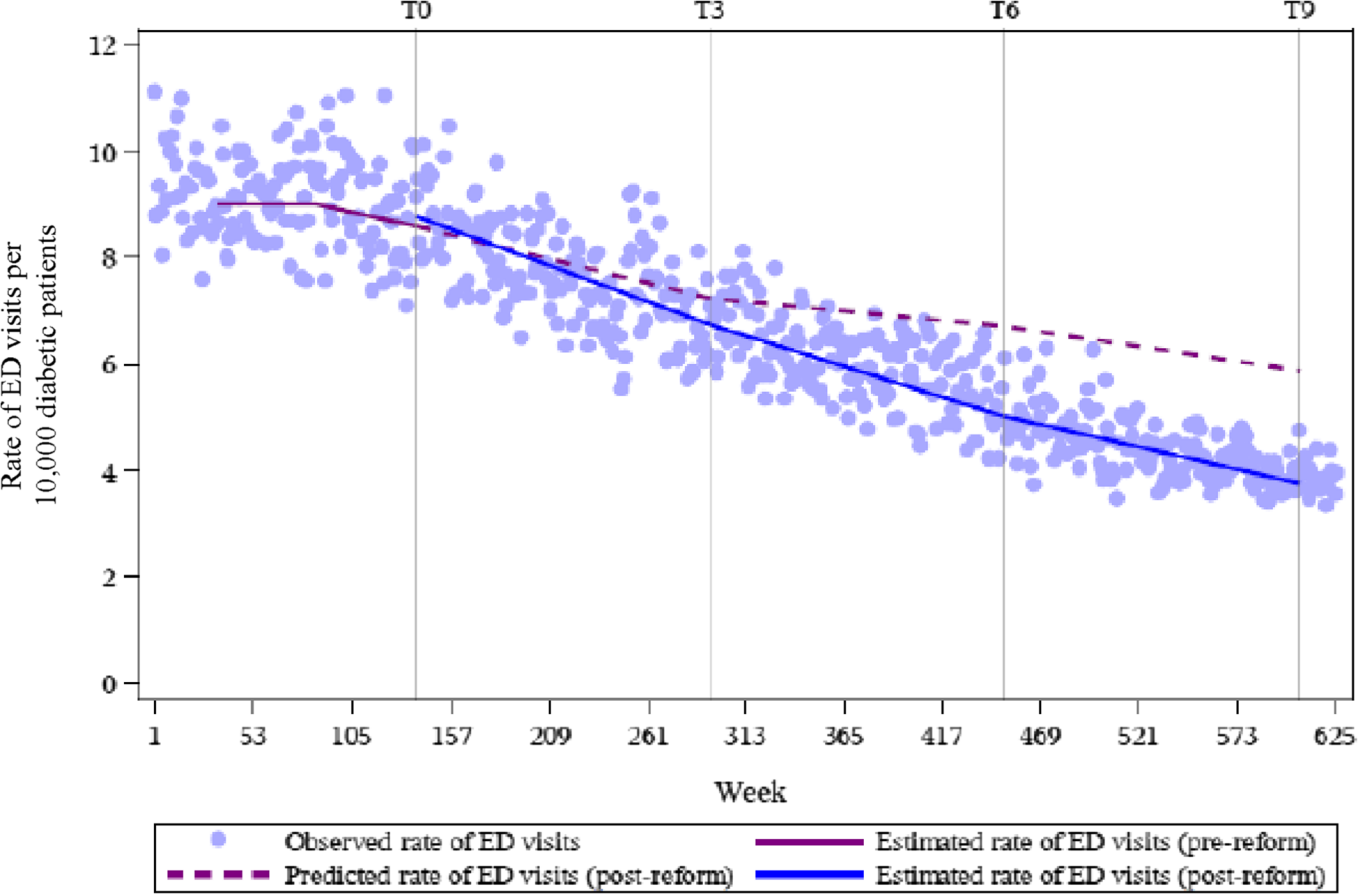
Weekly time series of the rate of avoidable ED visits among diabetic patients in Quebec in urban regions, 2000/01 to 2011/12

**Fig. 3:**
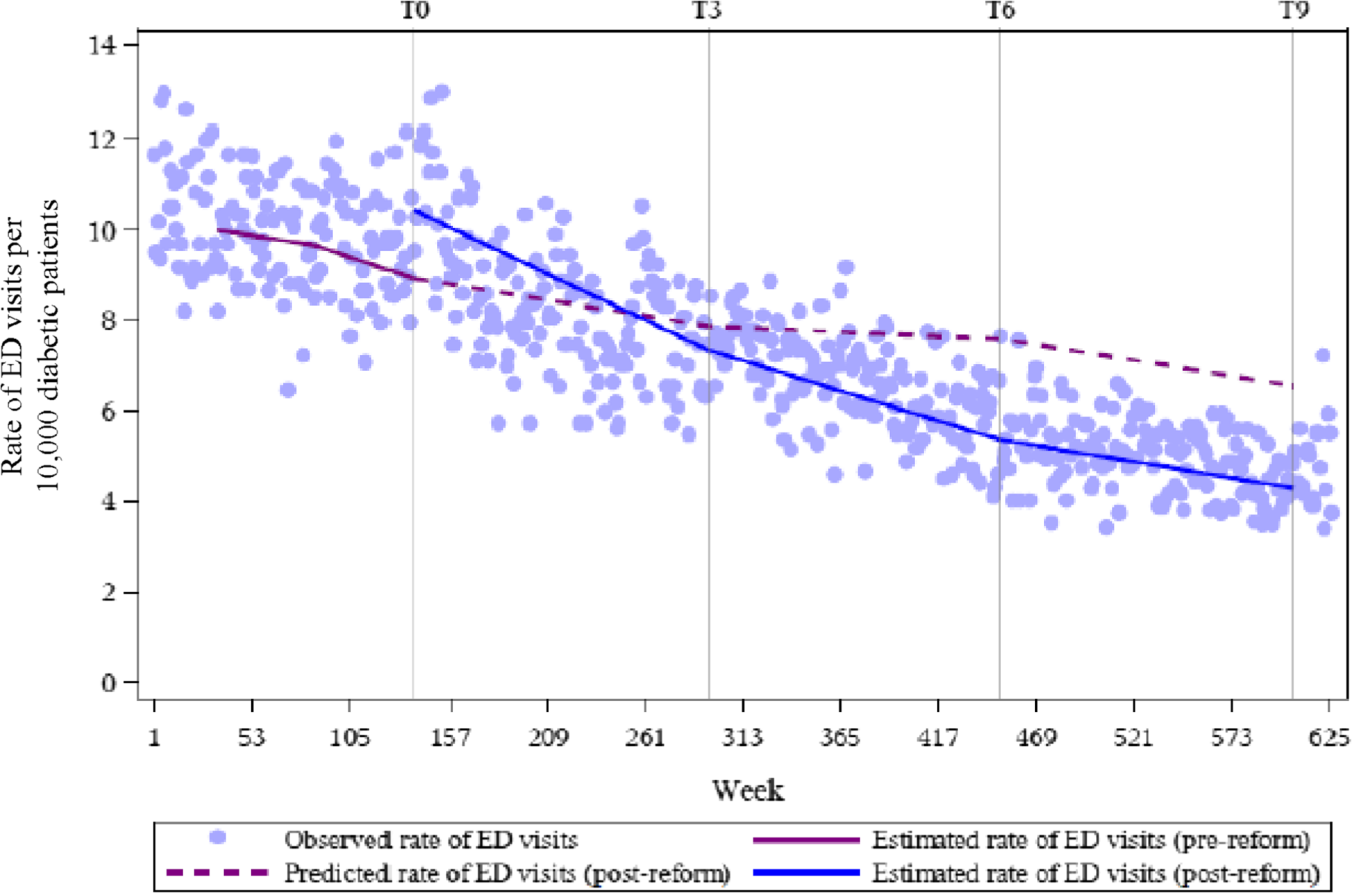
Weekly time series of the rate of avoidable ED visits among diabetic patients in Quebec in rural regions, 2000/01 to 2011/12

**Fig. 4:**
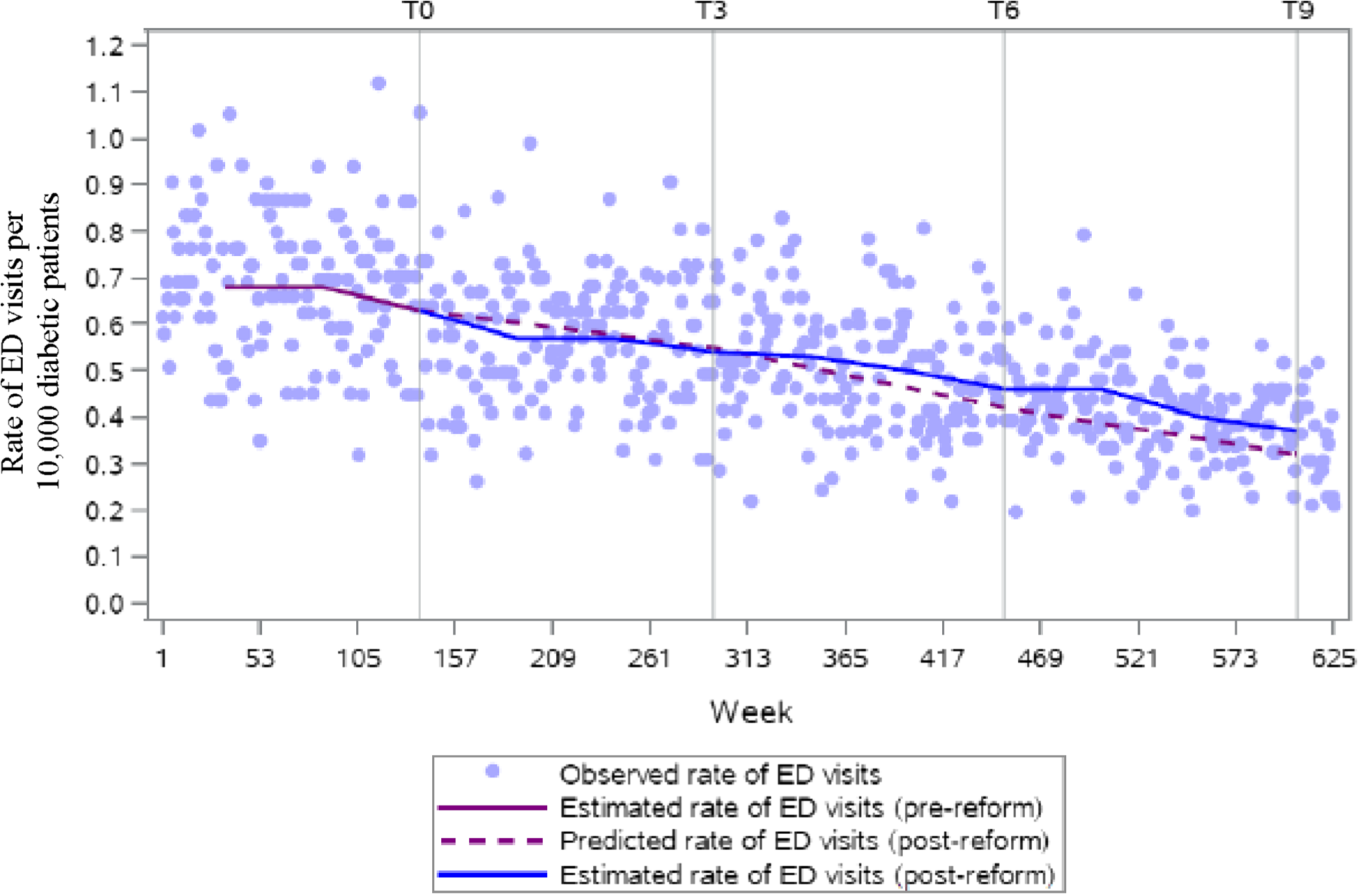
Weekly time series of the rate of ED visits among diabetic patients for appendicitis in Quebec, 2000/01 to 2011/12

Estimates from the regression model indicate decreases in the weekly rate of avoidable ED visits among diabetic patients. To contextualize these results, we calculated the difference between the estimated and predicted rates of avoidable ED visits, expressed as the number of avoidable visits to the ED per 10,000 diabetic patients per week, at distinct time points in the post-reform period. Table 2 quantifies the differences observed between the estimated and predicted rates in Figs. 2, 3 and 4. For acute diabetes-related visits, we found decreases in the rates for all time points. The differences between the estimated and predicted weekly rates were non-significant at 3 years post-reform in both urban and rural regions, respectively: −0.50 (95 % CI = −1.23, 0.23) and −0.54 (95 % CI = −1.83, 0.75). From *T*_*6*_ onwards, the differences between the estimated and predicted rates were significant in both regions. A statistically significant reduction of 2.12 (95 % CI = −2.94, −1.29) and 2.25 (95 % CI = −3.64, −0.85) cases per 10,000 diabetic patients per week was observed in urban and rural regions at *T*_*9*_, respectively. This meant that at *T*_*9*_, 469 weeks post-intervention, there was a difference of 2.12 visits per 10,000 diabetic patients per week between the estimated and predicted rates. The incremental decrease from *T*_*3*_ to *T*_*9*_ was roughly similar between urban and rural areas. For the appendicitis series, the differences between estimated and predicted rates were consistently non-significant up to and including *T*_*9*_ (RR_*T9*_ = 0.05; 95 % CI = −0.30, 0.40).

**Table 2:** Results for the rates of emergency department use for acute diabetes-related complications and appendicitis

Number of years since reform implementation	Rate of avoidable ED visits (per 10,000, per week)	Rate of ED visits for appendicitis (per 10,000, per week)
	Absolute change between the estimated and predicted rate post-reform (95 % CI)	Absolute change between the estimated and predicted rate post-reform (95 % CI)
Urban		
3	−0.50 (−1.23, 0.23)	−0.01 (−0.55, 0.53)
	*p* = 0.17	*p* = 0.97
6	−1.69 (−2.23, −1.14)	0.04 (−0.99, 1.07)
	*p* < 0.05	*p* = 0.95
9	−2.12 (−2.94, −1.29)	0.05 (−0.30, 0.40)
	*p* < 0.05	*p* = 0.79
Rural		
3	−0.54 (−1.83, 0.75)	
	*p* = 0.41	
6	−2.23 (−3.56, −0.89)	
	(*p* < 0.05	
9	−2.25 (−3.64, −0.85)	
	*p* < 0.05

We also assessed whether the increases in the magnitude of the differences between the estimated and predicted rates in urban and rural areas were statistically significant over time from *T*_*3*_ to *T*_*6*_ and from *T*_*6*_ to *T*_*9*_. Table 3 presents results for the urban, rural and appendicitis series. For all outcomes, these revealed non-significant changes in the absolute reduction of avoidable ED visits over the follow-up period.

**Table 3:** Absolute change between estimated and predicted differences between T_3_ and T_9_

Outcome	Time point	Absolute change (95 % CI)
Avoidable ED visits (urban)	*T*_*3*_ to *T*_*6*_	−1.19 (−2.95, 0.57)
	*T*_*6*_ to *T*_*9*_	−0.43 (−2.35, 1.49)
Avoidable ED visits (rural)	*T*_*3*_ to *T*_*6*_	−1.69 (−3.53, 0.15)
	*T*_*6*_ to *T*_*9*_	−0.02 (−1.94, 1.90)
Appendicitis	*T*_*3*_ to *T*_*6*_	0.03 (−1.04, 1.11)
	*T*_*6*_ to *T*_*9*_	−0.01 (−0.98, 0.96)

### Sensitivity analysis

Given that the rate of ED visits was modeled as a function of time before and after the introduction of the reform, an important consideration was our approach to modeling time trends. The segmented regression analysis assumes that the intervention produced a sudden change in the rate of avoidable visits to the ED. To explore whether our results were sensitive to how our main model was specified, we modeled the effect of the reform using a linear spline with a knot located at the point when the FMG model was introduced. The results were similar to those produced by the segmented regression. In urban areas, there was no change in the weekly rate of avoidable ED visits per 10,000 diabetic patients in the pre-reform period (RR = 1.00; 95 % CI = 0.99, 1.00). In the post-reform period, there was a significant 1 % decrease in the weekly rate of avoidable ED visits per 10,000 diabetic patients (RR = 0.99; 95 % CI = 0.98, 0.99). Similar results were observed for the rural regions. In each model for the urban and rural areas, the estimates for the pre and post reform slopes were significantly different from one another. There was no change in the weekly rate of visits to the ED for appendicitis per 10,000 diabetic patients in the pre-reform period (RR = 1.00; 95 % CI = 0.99, 1.01) or the post-reform period (RR = 1.00; 95 % CI = 0.99, 1.01).

## Discussion

### Interpretation

Our results indicate that the introduction of the FMG model produced reductions in the weekly rate of avoidable visits, suggesting some features of the FMG model are conducive to either increasing quality of or access to primary care that in turn contribute to less ED use. In comparison to other Canadian provinces, primary care reform in Quebec did not result in an overwhelming restructure of services. A 1 % decrease in the weekly rate of avoidable ED visits per 10,000 diabetic patients suggests that a greater reduction could be possible under a more encompassing reform. To place our results in context, a previous study found a 7 % reduction in the rate of total ED use in the first 3 years of the FMG reform among vulnerable patients who were enrolled with a physician practicing in a FMG versus those that were not [22].

The increase in the rate of avoidable visits in rural regions in the weeks closely following the introduction of the reform could be explained if FMG clinics do indeed increase access to primary care and therefore address previous unmet needs. While this would likely have a greater effect on the use of primary care services versus the ED, it is common in rural regions in Quebec for the ED to be used as a source of ambulatory care consultation. In this context, greater access to primary care may have resulted in greater diabetes screening or management in the ED based on pre-existing patterns of health service utilization.

Our model suggests that the magnitude of the changes between time points were not statistically significant from *T*_*3*_ onwards. This is evidenced by the non-significant post-reform slope (β_3_ in Table 1) and the absolute changes presented in Table 3. Between 2006 and 2011, the percentage of the population registered with a general practitioner in an FMG rose from 14 to 33 % [23]. Our findings imply that despite a greater proportion of the diabetes population being enrolled with FMG physicians across the province over time, the added benefit with regard to ED use may be minimal. A recent report released by the Auditor General in Quebec revealed shortcomings with how FMG practices have been implemented [24]. In particular, the report highlighted problems related to the management of centralized waiting lists for patients without a family doctor, and the contracts between health and social services agencies and FMG clinics on their territories that fail to adequately enforce the reform model in a notable number of practices [24]. Studies on primary care reforms in Quebec and Ontario have shown that physicians who decide to join new models are different from those who do not [2, 22, 25–29]. In this context, it would also be relevant to examine whether early adopters, who may be innovators in their field, implement it differently from those who join later. Future studies should account for the time-varying nature of the expansion of the FMG model across the province to determine whether there is an indication of a saturation effect. This may be important for informing policy responses that align with health system objectives.

On the spectrum of reforms to primary care in Canada, the FMG model is low-intensity in comparison to changes adopted in other provinces (e.g. blended forms of remuneration within team-based practices in certain primary care models in Ontario) [4]. Insofar that we can talk of an infrastructure for primary care in Quebec, the FMG model has the potential to be scaled-up and incorporate approaches to practice that facilitate chronic disease management using existing team-based inter-disciplinary care as a starting point [30]. Previous studies have suggested the potential for a chronic care model to reduce costs by decreasing hospitalizations and ED use among diabetic patients [31, 32].

### Strengths and limitations

Our use of administrative data to conduct an interrupted regression analysis that measured the reform’s effect on both quality of and access to primary care is relevant for policymakers, physicians, and to the development of diabetes surveillance in Quebec. Previous studies have provided important insight on issues of access, unmet need and patient experiences with primary care in the province using patient surveys [5, 33–35], and characteristics of care that are associated with ED use [36, 37]. We aimed to contribute to this literature by conducting a province-wide study that examines the effect of the reform over time using health administrative data. However, we expect that some visits for diabetes-related ACSCs were missed if the specific ICD-9 code used for outcome ascertainment was not recorded in the database. Under-counting visits to the ED would dampen any change that occurred due to the reform and thus produce an under-estimate of its effect.

The QICDSS contains administrative data from 1996 onwards and applies a previously validated 4-year clearance period before distinguishing between incident and prevalent cases of diabetes [17, 38]. As such, our study contained 2.6 years of pre-reform data starting in April 2000. The relatively short pre-reform period may affect our extrapolated rate estimation if the pre-reform trend in the 137 weeks leading up to the reform is not representative of the general trend in the years preceding the change. Booth et al. [39] showed that the rate of ED visits for acute diabetes-related complications in Ontario was declining during the 1990s, suggesting that improved glycemic control in primary care was an important contributing factor. The downward trend we observed prior to the FMG reform in Quebec implies a similar process. We find statistically significant differences in our main outcomes series after controlling for confounding and non-statistically significant results in our control series, which provides support to assertions that the reform contributed to additional rate decreases over time.

This was a physician-led reform and therefore take-up of the FMG model was, and remains, voluntary with staggered implementation across regions and time. Factors such as concurrent reforms are potential threats to study validity. A limitation of this study was the extent to which we could separate the effects of changes to the organization of primary care (the FMG model) from the effects of other changes that specifically targeted pathways of care to emergency health services. Caution when interpreting results from long post-reform periods is required since delayed and time-varying effects can complicate the analysis [40]. Given that we have 9 years of post-intervention follow-up, we cannot discount that other factors contributed to the effects we observed. It is possible that the effects observed 9 years after the introduction of the reform also include cumulative effects of other post- reform initiatives.

We attempted to address this concern using visits to the ED for appendicitis as a negative control series that would not be associated with the FMG reform yet is still sensitive to changes in the health system that are also related to our main outcome series. For instance, network clinics were introduced to facilitate the creation of a service corridor between primary care practices and specialized care in hospitals for a given territory [41]. Among the main objectives of the network clinics was to divert patient pathways of care away from the ED by facilitating access to diagnostic and imaging tests for individuals with chronic diseases [42]. CT scans are increasingly used to reduce negative appendectomy rates by improving the diagnosis of appendicitis, particularly among patients where clinical presentation is unclear. This is relevant for diabetic patients where acute abdominal pain can be attributed to other sources of gastrointestinal discomfort [43]. While the rate of visits to the ED for appendicitis would not be affected by the organization of primary care (FMG reform), it would potentially be sensitive to changes that offer patients an alternative entry point for accessing specialized hospital services. Although the negative control outcome is unlikely to account for all residual confounding, non-statistically significant post-reform trends (β_1_ + β_3_ in Table 1) and differences between the estimated and predicted rates from *T*_*3*_ to *T*_*9*_ in Table 2, lends support to our inferences that the FMG reform led to a decrease in ED visits.

This analysis used a single point in time to define the post-reform period. Although the policy was introduced in 2002, it was rolled-out over time as more physicians joined the model. Consequently, despite our use of a control series we cannot rule out the possibility of bias due to concurrent interventions. For instance, in 2008 a centralized waiting list for individuals without family physicians was introduced which may have also contributed to reduced use of the ED. A multiple baseline analysis that incorporated a different *T*_*0*_ for the year each practice switched to FMG status would have provided an added safeguard against bias. However this information was not available to us.

## Conclusion

Our findings suggest that the introduction of the FMG model produced a reduction in the weekly rate of avoidable visits to the ED. In extending this research further, it would be useful to assess relevant dimensions of access to and quality of care separately for diabetes and other conditions that are largely managed in primary care.

Our results also imply that despite a greater proportion of the diabetes population being enrolled with FMG physicians across the province over time, the added benefit may be minimal. Future studies should account for the time-varying nature of the expansion of the FMG model across the province and whether there is indication of a saturation effect. Evidence of this may suggest that FMG implementation differs according whether physicians adopted the model in the early or late post-reform reform period. This in turn may be important for policy makers if these groups systematically differ from each other. These considerations are applicable to other contexts in which reform uptake is voluntary and diffused over time.

## Abbreviations

ACSC: ambulatory care sensitive condition
ED: emergency department
FMG: family medicine group
ICD-9: international classification of disease, ninth revision
QICDSS: Quebec integrated chronic disease surveillance system

## References

[1] Clair M (2000). Rapport et recommendations: Solutions émergentes.

[2] Coyle N (2014). Characteristics of physicians and patients who join team-based primary care practices: Evidence from Quebec’s Family Medicine Groups. Health Policy.

[3] Ministère de la santé et des services sociaux (2002). Devenir un GMF: Guide d'accompagnement. From: http://www.csssqn.qc.ca/fr/emplois/examens-preparatoires-gmf-crq/guided-accompagnement-devenir-un-groupe-de-medecine-de-famille-gmf.download. Accessed September 20, 2014.

[4] Levesque J-F (2012). Looking Backward to Move Forward: A Synthesis of Primary Health Care Reform Evaluations in Canadian Provinces.

[5] Provost S (2010). Does Receiving Clinical Preventive Services Vary across Different Types of Primary Healthcare Organizations? Evidence from a Population-Based Survey. Healthc Policy.

[6] Commissaire à la santé et au bien-être (2009). Rapport d’appréciation de la performance du système de santé et de services sociaux du Québec 2009. Construire sur les bases d’une première ligne de soins renouvelée: Recommandations, enjeux et implications.

[7] Émond V (2002). Prévalence du diabète au Québec et dans ses régions: premières estimations d’après les fichiers administratifs.

[8] Pigeon É, Larocque I. Tendances temporelles de la prévalence et de l'incidence du diabète, et mortalité chez les diabétiques au Québec, de 2000-2001 à 2006-2007. 2011. From: https://www.inspq.qc.ca/pdf/publications/1239_TendancesDiabete2000-2001A2006-2007.pdf. Accessed November 15, 2013.

[9] Jaakkimainen L, Shah B, Kopp A, Hux J (2003). Sources of physician care for people with diabetes. Diabetes in Ontario: An ICES Practice Atlas.

[10] Clement M (2013). Canadian Diabetes Association 2013 Clinical Practice Guidelines for the Prevention and Management of Diabetes in Canada: Organization of Diabetes Care. Can J Diabetes.

[11] Imran SA, Rabasa-Lhoret R, Ross S (2013). Canadian Diabetes Association 2013 Clinical Practice Guidelines for the Prevention and Management of Diabetes in Canada: Targets for Glycemic Control. Can J Diabetes.

[12] Bernard LD (2013). Canadian Diabetes Association 2013 Clinical Practice Guidelines for the Prevention and Management of Diabetes in Canada: Monitoring Glycemic Control. Can J Diabetes.

[13] Wagner AK (2002). Segmented regression analysis of interrupted time series studies in medication use research. J Clin Pharm Ther.

[14] Ministère de santé et des services sociaux, Équipe d’évaluation des GMF (2008). Évaluation de l’implantation et des effets des premiers groupes de médecine de famille au Québec.

[15] Bindman AB (2005). The impact of Medicaid managed care on hospitalizations for ambulatory care sensitive conditions. Health Serv Res.

[16] Blais C (2014). Quebec Integrated Chronic Disease Surveillance System (QICDSS), an innovative approach. Chronic Dis Inj Can.

[17] Hux JE (2002). Diabetes in Ontario Determination of prevalence and incidence using a validated administrative data algorithm. Diabetes Care.

[18] Ouhoummane N (2010). Impact du diabète sur la mortalité à la suite d’une hospitalisation pour un premier infarctus aigu du myocarde au Québec. Département de médecine sociale et préventive.

[19] Santé et services sociaux Québec (2009). Focus on the Québec health and social services system.

[20] Canadian Institute for Health Information. Technical note: Ambulatory care sensitive conditions (ACSC). 2014 April 2015]; Available from: http://www.cihi.ca/CIHI-ext-portal/internet/en/document/health+system+performance/indicators/health/tech_acsc_2011. Accessed April 20, 2015.

[21] Belzile E, Sanche S, McCusker J, et al. A measure of emergency department use based on Quebec's administrative data 2011. From:http://smhc.qc.ca/ignitionweb/data/media_centre_files/490/A%20Measure%20of%20Emergency...%20EN_May%202011.pdf. Accessed October 15, 2013.

[22] Héroux J (2014). Marginal structural models for skewed outcomes: Identifying causal relationships in health care utilization. Stat Med.

[23] Commissaire à la santé et au bien-être. Assurer l'inscription de la population qui le désire auprès de groupes de médecins de première ligne 2011. From: http://www.csbe.gouv.qc.ca/fileadmin/www/2011/InfoPerformance/CSBE_Info_Performance_Bulletin_no2.pdf. Accessed April 20, 2015.

[24] Vérificateur général du Québec. Groupes de médecine de famille et cliniques-réseau 2015. From: http://www.vgq.gouv.qc.ca/fr/fr_publications/fr_rapport-annuel/fr_2015-2016-VORPrintemps/fr_Rapport2015-2016-VOR-Chap05.pdf. Accessed June 25, 2015.

[25] Kantarevic J, Kralj B, Weinkauf D (2011). Enhanced fee-for-service model and physician productivity: Evidence from Family Health Groups in Ontario. J Health Econ.

[26] Kantarevic J, Kralj B (2013). Link between pay for performance incentives and physician payment mechanisms: Evidence from the diabetes management incentive in Ontario. Health Econ..

[27] Kantarevic J, Kralj B (2014). Risk selection and cost shifting in a prospective physician payment system: Evidence from Ontario. Health Policy.

[28] Kantarevic J, Kralj B. Physician Payment Contracts in the Presence of Moral Hazard and Adverse Selection: The Theory and Its Application in Ontario. Health Economics, 2015. http://onlinelibrary.wiley.com/doi/10.1002/hec.3220/epdf. 10.1002/hec.3220 26239311

[29] Kralj B, Kantarevic J (2013). Quality and quantity in primary care mixed-payment models: Evidence from family health organizations in Ontario. Can J Econ.

[30] Coleman K (2009). Evidence On The Chronic Care Model In The New Millennium. Health Aff.

[31] Bodenheimer T, Wagner EH, Grumbach K (2002). Improving primary care for patients with chronic illness: the chronic care model, Part 2. JAMA.

[32] Wagner EH (2001). Effect of improved glycemic control on health care costs and utilization. JAMA.

[33] Levesque JF (2012). Emerging organisational models of primary healthcare and unmet needs for care: insights from a population-based survey in Quebec province. BMC Fam Pract.

[34] Pineault R (2011). Accessibility and Continuity of Care: A Study of Primary Healthcare in Quebec.

[35] Pineault R (2011). The influence of primary health care organizational models on patients’ experience of care in different chronic disease situations. Chronic Dis Inj Can.

[36] McCusker J (2010). Emergency department visits and primary care among adults with chronic conditions. Med Care.

[37] McCusker J (2012). Factors predicting patient use of the emergency department: a retrospective cohort study. CMAJ.

[38] Asghari S (2009). Optimal strategy to identify incidence of diagnostic of diabetes using administrative data. BMC Med Res Methodol.

[39] Booth GL (2005). Time trends and geographic disparities in acute complications of diabetes in Ontario, Canada. Diabetes Care.

[40] Salway R, Sims M, Gilmore AB (2014). Interpreting long-term trends in time series intervention studies of smoke-free legislation and health. Int J Stat Med Res.

[41] Ministère de la santé et des services sociaux (2013). Rapport annuel de gestion 2012–2013.

[42] Ministère de la santé et des services sociaux (2008). Orientations pour le développement des GMF et Cliniques réseau intégrés.

[43] Tsai S-H (2008). Complicated acute appendicitis in diabetic patients. Am J Surg.

